# Mycoplasma pneumoniae-Induced Rash and Mucositis: How Can We Help?

**DOI:** 10.7759/cureus.83794

**Published:** 2025-05-09

**Authors:** Rafaela D Gonçalves, Rita Carvalho, Marisa S Nunes, Inês Ferreira

**Affiliations:** 1 Pediatrics, Hospital de São Bernardo, Unidade Local de Saúde da Arrábida, Setúbal, PRT

**Keywords:** mirm, mycoplasma pneumoniae induced rash and mucositis, pediatric dermatology, pediatric infectious disease, rare skin disease, skin lesions

## Abstract

*Mycoplasma pneumoniae*-induced rash and mucositis (MIRM) is a rare clinical pathology distinct from erythema multiforme major and Stevens-Johnson syndrome. We present a case of a patient who developed severe oral mucositis following an *M. pneumoniae* respiratory tract infection. We aim to raise awareness of MIRM, its varying presentations, and its management, particularly in light of the limited scientific evidence and the variability in treatment strategies depending on disease severity.

## Introduction

*Mycoplasma pneumoniae* is a common cause of respiratory tract infections affecting children and young adults more commonly [1]. Moreover, it can lead to extrapulmonary manifestations in approximately 25% of patients [1]. These extrapulmonary presentations can affect multiple organ systems, including gastrointestinal, cardiovascular, hematological, dermatological, and even neurological [2].

Regarding dermatological manifestations, it can range from a mild rash to severe blistering [3]. *M. pneumoniae*-induced rash and mucositis (MIRM) is a recently recognized syndrome that was previously considered part of the Stevens-Johnson syndrome (SJS) and erythema multiforme (EM) spectrum. The presentations of MIRM vary and can be divided into specific categories according to cutaneous and mucosal involvement: classic MIRM, MIRM sine rash, and severe MIRM [3]. Compared to SJS and EM, MIRM primarily affects mucosal tissues, rarely involves hepatic or renal systems, and tends to have a relatively better prognosis [4]. This article describes a case of MIRM with distinct features, emphasizing its clinical course, diagnostic criteria, complications, and treatment options.

## Case presentation

**Figure 1:**
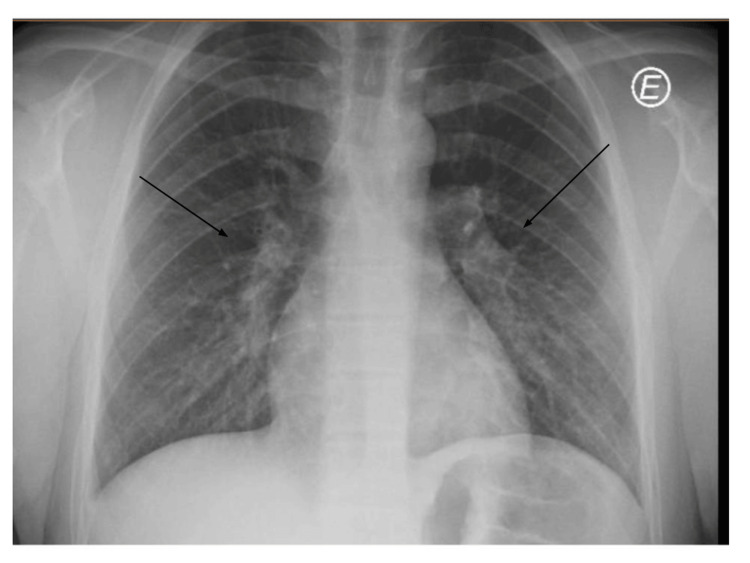
Chest X-ray on day 8 of disease (first admission in the ED) The X-ray reveals diffuse, patchy interstitial infiltrates scattered on both lungs.

A 14-year-old male, weighing 80 kg, with no significant past medical history, presented to our pediatric ED with a fever every four hours, with a maximum temperature of 40°C, associated with cough, sore throat, fatigue, and myalgias lasting for eight days. In the ED, a chest X-ray revealed bilateral interstitial infiltrates (Figure 1), and laboratory tests showed elevated inflammatory markers, including a WBC count of 10.8 × 10³/µL (normal range: 4.5-11.4 × 10³/µL), a neutrophil count of 7.9 × 10³/µL (normal range: 1.5-8 × 10³/µL), and an elevated CRP level of 11.31 mg/dL (normal: <0.5 mg/dL). His brother presented with similar symptoms and was hospitalized with atypical pneumonia due to* M. pneumoniae*. The patient was diagnosed with atypical pneumonia and treated with azithromycin (10 mg/kg/day for five days).

**Figure 2:**
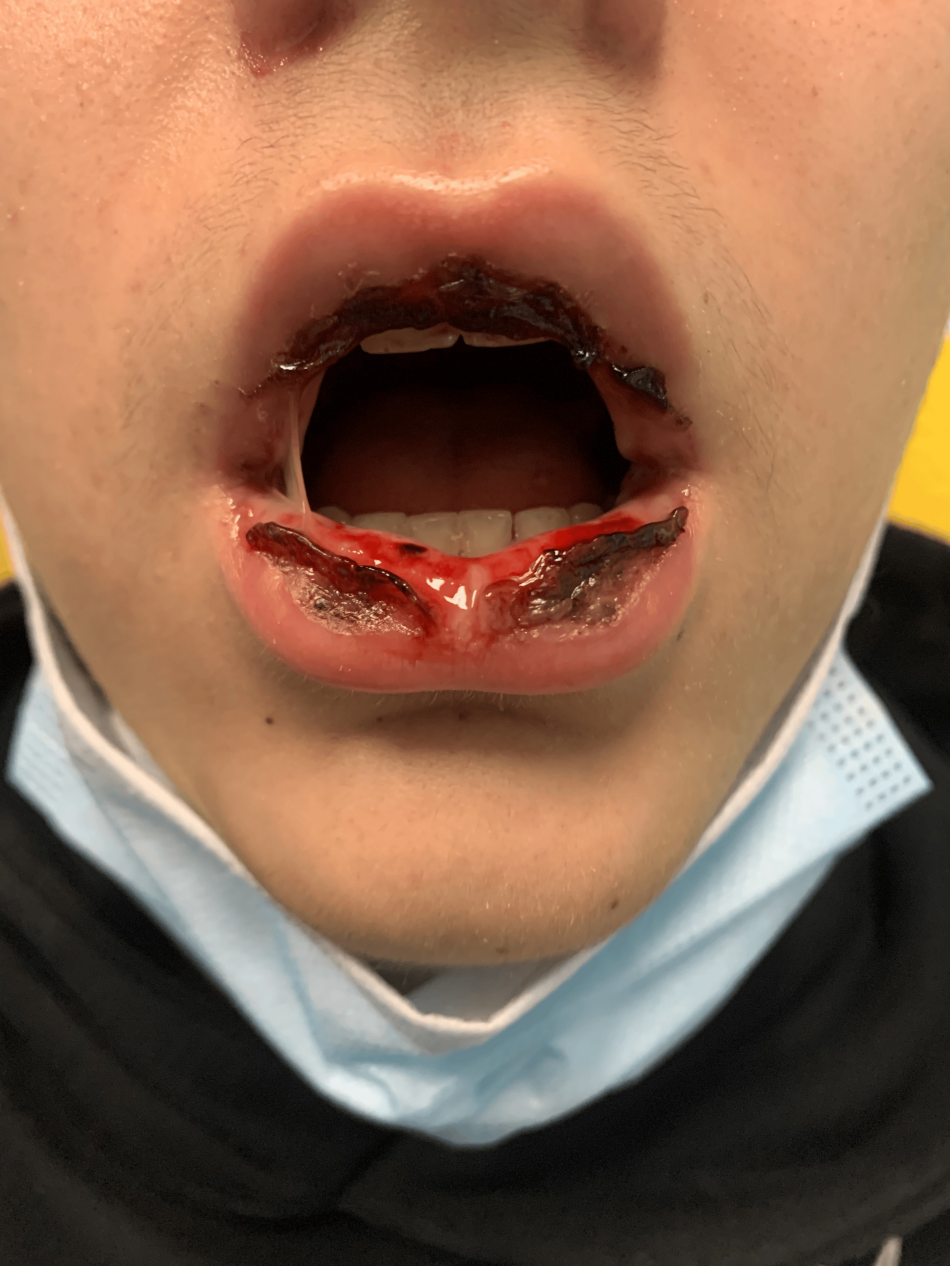
Clinical photograph showing erosions, confluent ulcers with crusts on the lips and mucosa

**Figure 3:**
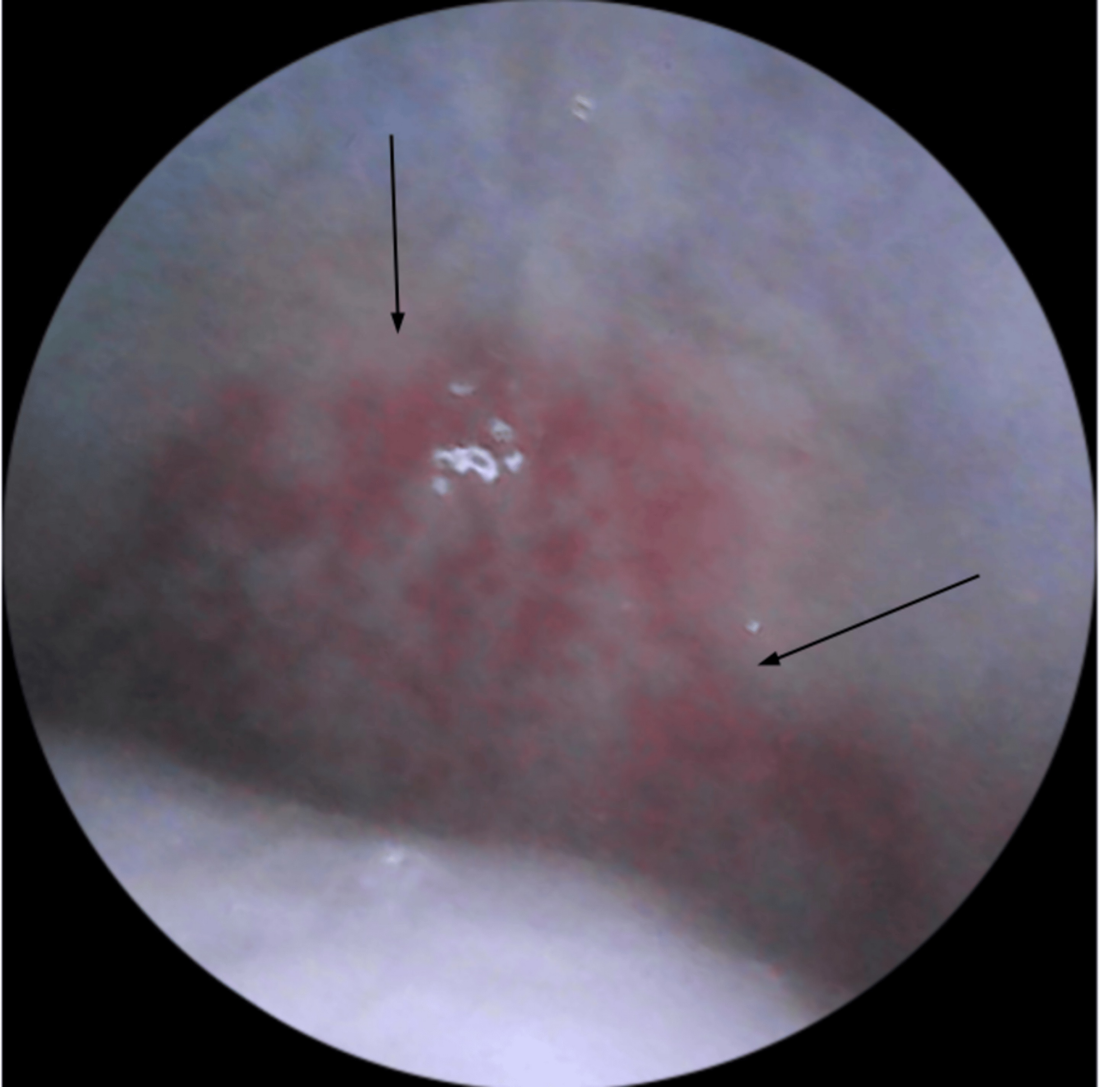
Clinical photograph showing soft and hard palate ulcerative lesions

**Figure 4:**
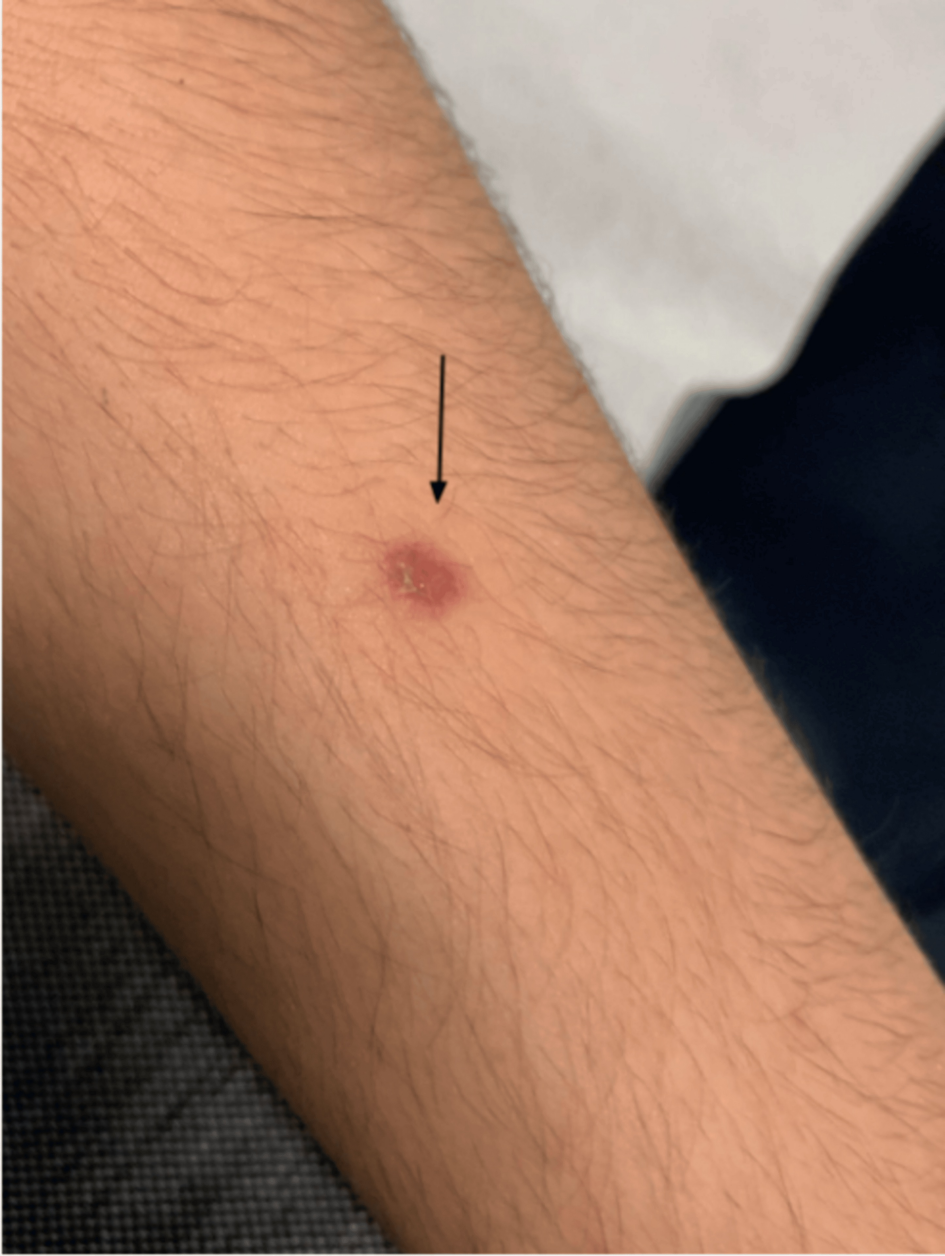
Clinical photograph showing a single erythematous papule on the left arm

On the fifth day of antibiotic therapy, the patient returned to the ED due to severe oral mucositis and inability to tolerate solids or liquids for the past two days. Upon admission, the patient was hemodynamically stable (blood pressure: 118/73 mmHg; heart rate: 65 bpm; SpO₂: 100%; body temperature: 36.4°C), mildly dehydrated, and had been afebrile for two days. Examination of the oral cavity revealed severe mucositis with hemorrhagic crusts on lips, vesiculobullous lesions on the buccal mucosa, soft palate, and hard palate (Figure 2, Figure 3). An eye examination revealed bilateral conjunctival hyperemia, and a skin examination revealed a single erythematous papule on the left arm (Figure 4). No lesions were found in the genital, anal, or perianal regions. Systemic and chest examinations revealed no significant findings, including the absence of hepatosplenomegaly, significant lymphadenopathy, or neurological abnormalities.

Laboratory tests revealed a WBC count of 10,500 cells/μL with 7.8 × 10³/µL neutrophils (normal range: 1.5-8 × 10³/µL) and an increased CRP level of 8.02 mg/dL, with normal renal function and no electrolyte imbalance. Serologies for Epstein-Barr virus, cytomegalovirus, herpes simplex virus types 1 and 2, as well as hepatitis B and C screening, were negative, and no immunological abnormalities were detected. Given the clinical context, syphilis testing was not performed for this adolescent at this time. PCR for herpesvirus and enterovirus in blood was negative, as was the multiplex PCR assay of a nasopharyngeal swab. Both IgM and IgG antibodies for *M. pneumoniae* were positive, with IgG levels exceeding 200 U/mL (positive: >10-20 U/mL). All results are summarized in Table 1. Based on these findings, a diagnosis of MIRM was made.

**Table 1:** Laboratory investigations and results * PCR multiplex respiratory panel: influenza A (including subtypes like H1N1), influenza B, respiratory syncytial virus (types A and B), adenovirus, parainfluenza virus (types 1, 2, 3, and 4, human metapneumovirus, rhinovirus/enterovirus, coronavirus (non-SARS-CoV-2 types, such as OC43, 229E, HKU1, and NL63), SARS-CoV-2, MERS - coronavirus, *Mycoplasma pneumoniae*, *Chlamydia pneumoniae*, *Bordetella pertussis*, and *Bordetella parapertussis* ** Multiplex PCR for herpesviruses and enteroviruses: HSV-1, HSV-2, varicella-zoster virus, human herpesvirus 6, enterovirus species A-D (coxsackieviruses and echoviruses), and poliovirus ALT, alanine aminotransferase; AST, aspartate aminotransferase; CMV, cytomegalovirus; EBV, Epstein-Barr virus; HCV, hepatitis C virus; HSV, herpes simplex virus

Investigation	Result	Normal range
Hematology		
Hemoglobin	15.5 g/dL	13-17 g/dL
Hematocrit	45.70%	40-50%
Leucocytes	10.5 × 10³/µL	4.5-11.4 × 10³/µL
Neutrophils	7.8 × 10³/µL	1.5 - 8.0 × 10³/µL
Lymphocytes	13.9 × 10³/µL	1.0 to 4.0 × 10³/µL
Renal function and electrolytes		
Creatinine	0.91 mg/dL	0.7-1.25 mg/dL
Urea	44 mg/dL	18-45 mg/dL
Sodium	141 mmol/L	136-146 mmol/L
Potassium	4 mmol/L	3.5-5.1 mmol/L
Liver enzymes		
AST	25 U/L	5-34 U/L
ALP	24 U/L	<55 U/L
Inflammatory marker		
CRP	8.02 mg/dL	<0.5 mg/dL
Infectious disease serology		
Hepatitis B surface antigen	Negative	-
Hepatitis B surface antibody	Negative	-
HIV 1/2 and p24 antigen	Negative	-
HCV	Negative	-
Multiplex PCR panels		
Respiratory panel*	Negative	-
Herpesviruses and enteroviruses**	Negative	-
*Mycoplasma pneumoniae* serology		
IgM for *M. pneumoniae*	Reactive	-
IgG for *M. pneumoniae*	Reactive (200 U/mL)	Negative <10 U/mL, positive >10 U/mL
CMV serology		
IgM for CMV	Nonreactive	Negative <0.7, equivocal 0.7-0.9, positive >0.9
IgG for CMV	Nonreactive	Negative <4, equivocal 4-6, positive >6
EBV serology		
Anti-EBNA-1 IgG	Reactive (588 U/mL)	Negative <5 U/mL, equivocal 5-20 U/mL, positive >20 U/mL
Anti-EBV-CA IgM	Nonreactive (<10 U/mL)	Negative <20 U/mL, equivocal 20-40 U/mL, positive >40 U/mL
Anti-EBV-CA IgG	Reactive (>750 U/mL)	Negative <20 U/mL, positive >20 U/mL
HSV serology		
IgG for HSV-1	Reactive (58.3 index value)	Negative <0.9, equivocal 0.9-1.1, positive >1.1
IgG for HSV-2	Nonreactive (<0.5 index value)	Negative <0.9, equivocal 0.9-1.1, positive >1.1
IgM for HSV-1/2	Nonreactive (<0.5 index value)	Negative <0.9, equivocal 0.9-1.1, positive >1.1
Parvovirus serology		
IgM for parvovirus	Nonreactive (<0.4 index value)	Negative <0.9, equivocal 0.9-1.1, positive >1.1
IgG for parvovirus	Nonreactive (<0.1 index value)	Negative <0.9, equivocal 0.9-1.1, positive >1.1

The patient was hospitalized and received supportive care, including IV fluids, topical treatment on mucous surfaces with nystatin, lidocaine, and sodium bicarbonate, as well as systemic analgesics. He was started on systemic corticosteroids, initially with IV methylprednisolone (1 mg/kg/day) for five days, followed by oral prednisolone (60 mg/day) for an additional three days. On the second day of hospitalization, the patient developed intense chest pain that worsened with food ingestion. Gastroenterology was consulted, and ulcerative lesions in the esophagus were suspected. The patient was started on esomeprazole (40 mg/day) and sucralfate (1 g before meals). Despite this treatment, the pain persisted, and IV morphine (0.05 mg/kg/day) was administered 30 minutes before meals, which improved both pain and oral intake. Morphine was discontinued after two days.

The patient was discharged after eight days, asymptomatic, with restored oral intake and no visible lesions. Ten weeks after discharge, the patient was reevaluated and found to be free of clinical symptoms.

## Discussion

MIRM primarily affects children and young adults, with a male predominance. Respiratory symptoms typically appear first, followed by mucocutaneous symptoms. There is usually a one-week interval between the two phases [3].

Although MIRM is usually a self-limited condition with a milder course than SJS/EM [1], it can still result in substantial complications and increased morbidity, often requiring hospitalization [1,2].

The underlying mechanisms of MIRM differ from those of SJS. A widely accepted hypothesis is that MIRM arises from the production of immunoglobulins due to the expansion of B cell clones, leading to the deposition of immune complexes within mucocutaneous tissues, which causes subsequent tissue damage. This contrasts with the type IV delayed hypersensitivity mechanism that underpins both EM and SJS [1,2], a distinction that has implications for treatment strategies.

Lesions associated with MIRM may present in diverse forms, including vesiculobullous, targetoid, atypical target, or occasionally maculopapular lesions, and they may involve the extremities, trunk, and sometimes the face [1]. Severe oral mucositis is a hallmark feature, typically presenting as hemorrhagic crusting on the lips and painful aphthae on the buccal mucosa and tongue [2].

According to Canavan et al., the diagnostic criteria for MIRM are explained in Table 2 [3].

**Table 2:** Diagnosis criteria for MIRM MIRM, *Mycoplasma pneumoniae*-induced rash and mucositis Source: Canavan et al. (2015) [3]; with permission from Elsevier

Criterion	Description
Evidence of atypical pneumonia	Fever, cough, positive auscultatory findings, and increased Mycoplasma pneumoniae IgM antibodies or detection of the pathogen in oropharyngeal swabs, bullae, PCR, and/or serial cold agglutinins
Limited skin detachment	Involvement of less than 10% of the body surface area
Mucosal involvement	Affects at least two mucosal sites
Skin lesions	Few vesiculobullous lesions or scattered atypical target lesions may be present

MIRM can be categorized into three types based on its cutaneous and non-mucosal rash patterns: classic MIRM, MIRM sine rash, and severe MIRM. All types meet the criteria for MIRM diagnosis above and are distinguished by the following characteristics (Table 3) [3].

**Table 3:** MIRM subtypes classification and description MIRM, *Mycoplasma pneumoniae*-induced rash and mucositis Source: Canavan et al. (2015) [3]; with permission from Elsevier

MIRM subtypes	
Classic MIRM	This form is marked by the presence of a non-mucosal rash that includes a variety of lesions, such as vesiculobullous (77%), scattered target lesions (48%), papules (14%), macules (12%), and morbilliform eruptions (9%).
MIRM sine rash	In this variant, there is minimal or no prominent cutaneous rash, though a small number of morbilliform lesions or vesicles might be observed.
Severe MIRM	This type involves more than two mucosal areas, along with a widespread cutaneous rash that includes extensive non-mucosal blisters or atypical flat target lesions.

In this case, our patient meets the criteria for MIRM sine rash.

Laboratory testing, including multiplex PCR assays and serum IgM/IgG titers, should be employed to confirm the diagnosis. However, these diagnostic tools have certain limitations. While PCR is significantly sensitive and specific, it can remain positive for up to four months post-infection, and it cannot differentiate between asymptomatic carriers and those with active infection [2,5]. Serological tests may yield negative results in the acute phase since IgM antibodies typically begin to increase about a week after infection, reach their zenith between three to six weeks, and can remain elevated for several months, while IgG titers reach their maximum level around two weeks and continue for years [2]. To achieve a more accurate diagnosis, both methods should be considered, as demonstrated in the current case, with results interpreted in the context of the clinical presentation, while accounting for the challenges related to methodology and sensitivity.

Currently, no specific, evidence-based therapy/guidelines exist for MIRM management. Thus, supportive care remains the keystone of treatment, aimed at relieving symptoms and preventing further complications. Antibiotic therapy, particularly with macrolides such as azithromycin, has been reported to be effective in managing MIRM. Azithromycin is frequently used worldwide due to its anti-inflammatory properties, which may be advantageous in cases where an immunological component is suspected [1]. In addition, azithromycin reduces the presence of* M. pneumoniae *in the respiratory tract, thereby alleviating excess antigenic stimulation [4].

In some cases, adjunctive therapies may enhance recovery and have been associated with positive outcomes in case reports. These include immunosuppressive and immunomodulatory treatments [2,6,7]. Corticosteroids have been shown to provide clinical benefit. A short course (five to 10 days) of prednisone at 1 mg/kg/day without taper has been suggested for patients with extensive mucosal involvement and severe symptoms. In severe cases, where oral administration is not tolerated, IV methylprednisolone may be used at a dosage of 1-2 mg/kg/day. [1]. Other treatments, such as IV immunoglobulin (IVIG) (0.5 g/kg/day for four days) [2,3] and cyclosporine A [8], have also been described in the literature. One case report demonstrated that IVIG was effective in treating MIRM when conventional therapies failed. However, the efficacy of each therapy remains uncertain, accentuating the need for further research into optimal treatment regimens [8]. In our case, adjunctive therapy was essential in the recovery process, leading to an excellent outcome.

The overall prognosis for MIRM is generally favorable, with low morbidity and mortality rates (around 3%). However, sequelae can still occur, particularly when there is extensive mucosal involvement [1]. Recurrence of MIRM is seldom, occurring in approximately 8% of cases [6].

The authors emphasize the importance of reporting additional MIRM cases to improve our understanding of its clinical manifestations, diagnostic approaches, and management strategies. Further research is essential to establish evidence-based guidelines for the most effective treatments.

## Conclusions

Early recognition of MIRM as a distinct condition from SJS and EM is crucial, as its unique pathophysiological mechanisms linked to *M. pneumoniae* infection directly influence treatment decisions. The variability in clinical presentations and diagnostic criteria makes diagnosis challenging, requiring careful attention. Identifying the causative agent is essential for an accurate diagnosis. Although treatment remains primarily supportive, immunomodulatory therapies may play an important role, especially in more severe cases.
